# Stable isotope compositions (δ^2^H, δ^18^O and δ^17^O) of rainfall and snowfall in the central United States

**DOI:** 10.1038/s41598-018-25102-7

**Published:** 2018-04-30

**Authors:** Chao Tian, Lixin Wang, Kudzai Farai Kaseke, Broxton W. Bird

**Affiliations:** Department of Earth Sciences, Indiana University-Purdue University Indianapolis (IUPUI), Indianapolis, IN 46202 USA

## Abstract

Stable isotopes of hydrogen and oxygen (δ^2^H, δ^18^O and δ^17^O) can be used as natural tracers to improve our understanding of hydrological and meteorological processes. Studies of precipitation isotopes, especially ^17^O-excess observations, are extremely limited in the mid-latitudes. To fill this knowledge gap, we measured δ^2^H, δ^18^O and δ^17^O of event-based precipitation samples collected from Indianapolis, Indiana, USA over two years and investigated the influence of meteorological factors on precipitation isotope variations. The results showed that the daily temperature played a major role in controlling the isotope variations. Precipitation experienced kinetic fractionation associated with evaporation at the moisture source in the spring and summer and for rainfall, while snowfall, as well as precipitation in the fall and winter, were mainly affected by equilibrium fractionation. The ^17^O-excess of both rainfall and snowfall were not affected by local meteorological factors over the whole study period. At the seasonal scale, it was the case only for the spring. Therefore, ^17^O-excess of rainfall, snowfall and the spring precipitation could be considered as tracers of evaporative conditions at the moisture source. This study provides a unique precipitation isotope dataset for mid-latitudes and provides a more mechanistic understanding of precipitation formation mechanisms in this region.

## Introduction

Stable hydrogen and oxygen isotopes (δ^2^H, δ^18^O and δ^17^O) can be used as powerful tracers to investigate hydrological processes across multiple spatial scales, including the ecohydrological (e.g., identification of vegetation water sources and partitioning of evapotranspiration) and hydroclimatic processes (e.g., separating hydrographs and quantifying atmospheric processes)^1–6^. The δ^2^H and δ^18^O variations of precipitation are mainly determined by temperature in middle and high latitudes^7^, while precipitation amount is the main determining factor in the tropics^8^. Variations in the isotopic composition of precipitation are also affected by the source of air masses, elevation of condensation, distance from the coast, and latitude^3,7^. In addition, for individual precipitation events at a site, the isotopic composition is influenced by synoptic weather patterns, such as the atmospheric conditions at the moisture source, moisture transport trajectories, mixing between vapors from different origins and subcloud processes (e.g., re-evaporation and convection)^2,3,9^.

During phase change (e.g., evaporation, condensation, and sublimation), two different types of mass-dependent fractionation process may occur between water vapor and condensed water (liquid or ice crystals)^7,10^. One is equilibrium fractionation, which is driven by the lower saturation vapor pressure of the heavy isotope molecules with respect to the light isotopes^11,12^. Liquid condensation is generally thought to be a near-equilibrium process that is controlled by local temperature alone^11^. The other is kinetic fractionation, which is caused by different diffusivities of different isotopes (i.e., isotopically light molecules diffuse faster than those that are isotopically heavier)^12,13^. Kinetic fractionation, on the other hand, is related to unidirectional and incomplete reactions^11^ involved in evaporation at the moisture source site, re-evaporation at the precipitation site and solid condensation at supersaturation with respect to ice crystals (e.g., snowflakes and ice formation)^7,13,14^.

Although individual stable isotope ratios (δ^2^H and δ^18^O) of precipitation are informative, a second-order isotopic variable, deuterium excess (d-excess = δ^2^H − 8 × δ^18^O)^15^, can be further utilized to constrain temporal and spatial variations in ecohydrological processes and hydroclimatic conditions^15–19^. The d-excess is less variable compared with the individual isotopes (δ^2^H or δ^18^O) during the equilibrium fractionation because co-variation of δ^2^H and δ^18^O is eliminated and it is more sensitive to the kinetic fractionation processes^2,20^. d-excess therefore provides an additional constraint on conditions at the moisture source and processes that occur along the vapor’s trajectory as it travels from its origin to the precipitation site, including evaporation at the moisture source, condensation in supersaturation conditions, re-evaporation of raindrops, and moisture exchange in the cloud and sub-cloud layer^2,7,9,19^.

^17^O, the least abundant stable isotope form of oxygen, holds potential to provide additional constraints on the mechanisms of precipitation formation. Recent developments of high-precision analytical methods (e.g., water fluorination technique) have made it possible to measure changes in ^17^O despite its low natural abundance. Like d-excess, δ^17^O and δ^18^O show different sensitivities to equilibrium and kinetic fractionation processes^13^, which has led to another second-order parameter, ^17^O-excess (^17^O-excess = ln (δ^17^O + 1) − 0.528 × ln (δ^18^O + 1))^21^, as a new hydrological tracer. In theory, the ^17^O-excess of precipitation is not influenced by moisture source temperature because of similar temperature effects on ^17^O and ^18^O^22–24^. Recent studies from Antarctica, however, show that ^17^O-excess during snow formation under extreme cold condition has a strong sensitivity to atmospheric temperature due to condensation in supersaturation conditions affected by kinetic fractionation^10,25,26^. Therefore, unlike the d-excess (sensitive to both temperature and relative humidity (RH))^27^, ^17^O-excess in precipitation is mainly affected by the RH and insensitive to temperature at the moisture source, though it may be affected by the supersaturation effect under extremely cold conditions (−80 to −15 °C)^25,28–30^. Thus far, the studies of ^17^O-excess variations in precipitation have mainly focused on high-latitude snow and ice cores^10,25,30,31^, tropical storms^8^, and tap water (used as a proxy of precipitation) across the continental United States (U.S.)^19^. There are a limited number of studies on the meteorological factors that influence precipitation isotope variations in the mid-latitude regions. Therefore, to fill the gap in global precipitation isotope datasets, especially for ^17^O-excess in the mid-latitude regions, we investigated precipitation isotope dynamics during different seasons and explored rainfall-snowfall variations at one site from the Midwestern U.S. To better understand the formation mechanisms of precipitation and expand the role of ^17^O-excess as a tracer in investigating various ecohydrological processes at different scales, we examined the relationships between ^17^O-excess, δ^18^O and d-excess, and analyzed the relationships between isotope variations and the meteorological factors at the local site.

## Materials and Methods

### Sampling site

Event-based precipitation samples were collected in Zionsville, Indiana (39.88°N, 86.27°W; 258 m above sea level). Mean annual temperature at the site averaged 10.2 °C, with minimum monthly average temperature in February (−7.2 °C), and maximum average monthly temperature in July (22.2 °C) based on meteorological data from 2014 to 2015 (https://www.wunderground.com). The mean annual precipitation was 953.3 mm, with over 54% of the precipitation occurring between April and July with the highest monthly precipitation occurring in June. Precipitation at the site is influenced by different water vapor sources (Continental, Pacific, Atlantic, Gulf of Mexico, and Arctic)^32–34^, leading to relatively complicated influencing factors of the precipitation formation. Based on the climatology of Indiana, a calendar year was divided into four seasons, with spring as March through May, summer as June through August, fall as September through November, and winter as December through February^35^.

### Precipitation sample collections

In this study, event-based precipitation samples were collected from June 2014 to May 2016. In total we collected 235 precipitation samples consisting of 201 rainfall events and 34 snowfall events. To reduce evaporation effects on isotopes, samples were transferred from the precipitation collector to sealed glass vials (Qorpak Bottles, Fisher Scientific Co. Germany) immediately after each event. The samples were then stored at 4 °C until isotope analysis. If the precipitation event was finished after midnight, sampling was conducted at the earliest possible time in the morning. Snowfall samples were melted in sealed plastic bags, poured into the vials and then stored. Prior to measurements, samples containing impurities were filtered with 0.45 μm syringe filters (Cellulose Nitrate Membrane Filters, GE Healthcare Co. UK) or centrifuged (Iec Centra CL2 Centrifuge, Thermo Electron Co. USA) depending on the size of the impurities.

### Isotope analysis

#### δ^2^H, δ^18^O and δ^17^O measurements

Isotopic ratios (δ^2^H, δ^18^O and δ^17^O) of all the precipitation samples were simultaneously measured using a Triple Water Vapor Isotope Analyzer (T-WVIA-45-EP; Los Gatos Research Inc. (LGR), Mountain View, CA, USA), which is based on Off-Axis Integrated Cavity Output Spectroscopy (OA-ICOS) technique, coupling with a Water Vapor Isotope Standard Source (WVISS, LGR, Mountain View, CA, USA) at IUPUI (Indiana University-Purdue University Indianapolis) Ecohydrology Lab. The specific operational procedure has been described by Tian *et al*.^36^, therefore only a brief overview was provided here. In order to achieve higher accuracy and precision, the internal temperature of T-WVIA and WVISS were preheated to 80 °C and 50 °C, respectively, and the Teflon tubing connecting the WVISS and the T-WVIA was heated using pipe-heating cable to avoid condensation of water vapor. According to our previous work^36^, the higher accuracy and precision of our instruments are generally observed under moderate water vapor concentrations (10000–15000 ppm) for all isotopes, so all the samples were measured under 13000 ppm. Each sample was measured for 2 minutes, and the data output frequency for water isotope measurements was 1 Hz, translating to 120 data points for each sample. To attain more accurate ^17^O-excess measurements, the 1-Hz data were not averaged over the 2-min interval, the detailed calculation procedure was shown in the “^17^O-excess data processing” section.

#### Isotope calibration and normalization

Five commercially available working standards from LGR with known isotopic composition, spanning the entire range of our sample measurements (−154.0‰ to −9.2‰, −19.49‰ to −2.69‰ and −10.30‰ to −1.39‰ for δ^2^H, δ^18^O and δ^17^O, respectively), were analyzed routinely as reference waters after every five precipitation samples to check the instrument performance. In addition, in order to reduce inter-laboratory difference using different technique and calibration methods, all of the isotope ratios were normalized using two international water standards Vienna Standard Mean Ocean Water (VSMOW) and Standard Light Antarctic Precipitation (SLAP) following the procedure described in Schoenemann *et al*.^37^:

1 $${\delta }_{sample/VSMOW-SLAP}^{normalized}={\delta }_{sample/VSMOW}^{measured}\frac{({\delta }_{SLAP/VSMOW}^{assigned})}{({\delta }_{SLAP/VSMOW}^{measured})}$$

where δ is the δ^2^H, δ^18^O or δ^17^O, and the assigned values of δ^2^H_SLAP/VSMOW_, δ^18^O_SLAP/VSMOW_ and δ^17^O_SLAP/VSMOW_ are −427.50‰, −55.50‰ and −29.6986‰, respectively. In our study, SLAP2 is used as the replacement water standard for SLAP, and it is not significantly different from SLAP for δ^18^O or δ^17^O^38^. Therefore, SLAP2 is still referred as SLAP hereafter. The two international standards (VSMOW and SLAP) were measured once during each day of the measurements.

#### ^17^O-excess data processing

Since ^17^O-excess measurements are two orders of magnitude smaller than traditional δ^18^O measurements (per meg, i.e., 0.001‰), small peculiarities in either δ^18^O or δ^17^O may result in significant ^17^O-excess error^30^. To ensure the accuracy of ^17^O-excess measurements, we used mass-dependent fractionation coefficient (θ = ln (δ^17^O + 1)/ln (δ^18^O + 1)), varying slightly depending on the degree fractionation processes, as a quality control filter to check each individual measurement. Based on previous studies, the fractionation coefficient of water was found to be 0.511 ± 0.005 for kinetic transport effects^1^ and 0.529 ± 0.001 for equilibrium effects^39^. In addition, it has been shown in previous studies that almost all of the ^17^O-excess values of global precipitation (e.g., rainfall, snowfall, and ice) fall within the range of −100 to + 100 per meg^8,9,19,25,29,31^. Therefore, in order to minimize sources of error, any measurements outside the 0.506 and 0.530 range, as well as outside the observed range (−100 to +100 per meg), were removed from the analysis. The final ^17^O-excess value for every precipitation sample was given as the mean value of quality-controlled data. Using this method, the precision of SLAP was 0.79‰, 0.04‰, 0.02‰ and 3 per meg for δ^2^H, δ^18^O, δ^17^O and ^17^O-excess, respectively. To check the stability of our instrument precision, we also measured the GISP (Greenland Ice Sheet Precipitation, an international standard) and five commercially available working standards from LGR as mentioned above (−154‰ to −9‰, −20‰ to −3‰ and −10‰ to −1‰ for δ^2^H, δ^18^O and δ^17^O, respectively) on the VSMOW-SLAP scale. The precision of these measurements was better than 0.80‰, 0.06‰, 0.03‰ and 12 per meg for δ^2^H, δ^18^O, δ^17^O and ^17^O-excess, respectively (Table S1).

δ^17^O measurements are typically performed using the fluorination method for IRMS technique^19,26,27,40^, and the water sample are repeatedly measured several times. In addition, when measuring δ^17^O using the Picarro L2140-i wavelength-scanned cavity ring-down spectroscopy (WS-CRDS) instrument (Picarro Inc., Sunnyvale, CA, USA) at the University of Bern CEP (Climate and Environmental Physics) station, only the last three values were used^9^. These studies reassure us that data quality control by filtering out measurements could be a suggested procedure for ^17^O-excess determination. Moreover, the ^17^O-excess precision of our OA-ICOS technique (2 to 12 per meg) is comparable with IRMS technique (4 to 13 per meg)^19,25,29,31,37,41^ and CRDS method (< 10 per meg)^9,42^.

Besides analyzing event-based isotope data, to be comparable with many global precipitation isotope data sets such as Global Network of Isotopes in Precipitation (GNIP), amount-weighted isotopic composition at monthly or seasonal scales was also used in this study, as shown in equation (2).

2 $${\delta }_{i}=\frac{{\sum }_{i=1}^{n}{\delta }_{i}{P}_{i}}{{\sum }_{i=1}^{n}{P}_{i}}$$

where ${\delta }_{{i}}$ is the isotopic composition of an individual precipitation event with precipitation amount of ${P}_{i}$, *n* is the total number of precipitation events in a month or within a season.

### Meteorological variables

In order to investigate the effects of meteorological factors on isotopic variations and examine the mechanisms of precipitation formation during different seasons, we used the temperature, RH and precipitation amount at the study site. The meteorological data during the study period were obtained from the Zionsville meteorological station (https://www.wunderground.com). In order to determine whether local meteorological factors affected the isotopic variations in a practical sense, we set the threshold of r being 0.32 (i.e., R^2^ > 0.10, *p* < 0.05).

### Data availability statement

The datasets generated from the current study are available from the corresponding author on reasonable request.

## Results and Discussion

### The characteristics of precipitation, temperature and RH

**Figure 1:**
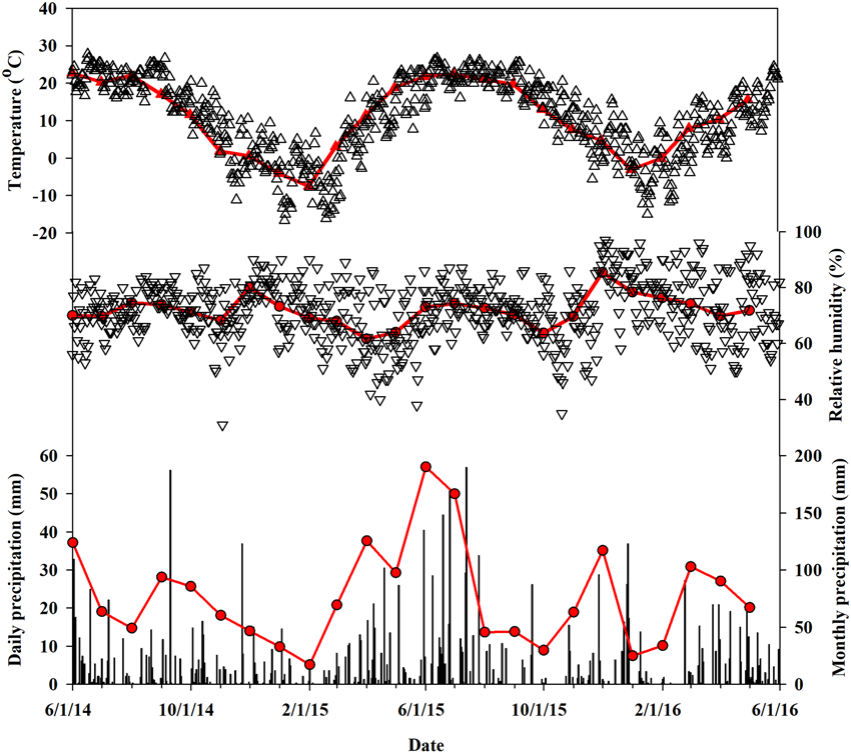
Daily (triangle and vertical bars) and monthly (red lines) precipitation, temperature and relatively humidity at Zionsville meteorological station between June 2014 and May 2016.

Figure 1 shows the daily and monthly meteorological characteristics (i.e., precipitation, temperature and RH) of the sample collection site between June 2014 and May 2016. All the meteorological variables showed distinct seasonal variations (Table 1). Precipitation amount and frequency were mainly concentrated in the summer (319 mm/35%), less in the spring and fall (276 mm/30% and 189 mm/20%, respectively), and the least in the winter (136 mm/15%). Average summer and winter temperatures were 21.7 °C and −1.6 °C, respectively. Temperatures in the spring (11.2 °C) and fall (11.9 °C) were similar. RH increased from 68.2% in the spring to 72.3% in the summer, then decreased to 69.5% in the fall and then reached a maximum of 77.1% in the winter.

**Table 1:** Monthly amount-weighted mean values of δ^2^H, δ^18^O, δ^17^O, ^17^O-excess and d-excess as well as monthly average temperature, relative humidity and total precipitation in central Indiana (June 2014-May 2016).

Date	δ^2^H (‰)	δ^18^O (‰)	δ^17^O (‰)	^17^O-excess (per meg)	d-excess (‰)	Temperature (°C)	Relative humidity (%)	Precipitation (mm)
Jun-14	−26.52	−4.20	−2.19	33	7.11	22.7	69.9	124.0
Jul-14	−31.91	−4.83	−2.53	26	6.72	20.3	69.7	63.5
Aug-14	−22.74	−3.58	−1.87	24	5.86	22.1	74.5	49.0
Sep-14	−28.87	−4.91	−2.56	36	10.39	17.1	73.9	93.7
Oct-14	−59.62	−8.51	−4.47	35	8.44	11.6	71.1	85.6
Nov-14	−72.96	−11.02	−5.80	34	15.18	1.9	68.2	60.2
Dec-14	−80.88	−11.77	−6.19	37	13.25	0.6	80.1	46.5
Jan-15	−84.82	−12.26	−6.47	26	13.25	−4.1	73.2	32.8
Feb-15	−143.38	−18.83	−9.96	32	7.29	−7.5	69.0	17.0
Mar-15	−87.04	−11.92	−6.28	33	8.30	3.0	68.0	69.3
Apr-15	−40.06	−5.96	−3.12	28	7.63	11.4	61.6	125.5
May-15	−15.98	−3.48	−1.80	36	11.86	18.8	63.9	97.5
Jun-15	−43.15	−6.29	−3.30	30	7.17	21.8	73.0	190.2
Jul-15	−25.38	−4.45	−2.32	35	10.25	22.4	74.3	166.6
Aug-15	−21.22	−3.81	−1.99	21	9.26	21.0	72.6	45.5
Sep-15	−26.31	−5.24	−2.72	44	15.58	19.8	70.3	46.0
Oct-15	−66.09	−10.46	−5.50	41	17.61	13.0	63.7	29.7
Nov-15	−57.68	−8.70	−4.57	33	11.92	7.7	69.6	63.0
Dec-15	−49.63	−7.45	−3.90	37	9.98	4.7	85.4	117.1
Jan-16	−71.88	−11.05	−5.82	33	16.53	−3.1	78.4	24.9
Feb-16	−87.70	−12.44	−6.54	41	11.78	−0.1	76.3	33.8
Mar-16	−32.14	−5.19	−2.72	26	9.39	7.9	74.2	102.9
Apr-16	−38.49	−5.54	−2.89	35	5.83	10.4	69.8	90.2
May-16	−31.89	−4.68	−2.45	28	5.55	15.6	71.8	67.1
Spring	−38.98	−5.90	−3.09	31	8.24	11.2	68.2	276.3
Summer	−31.04	−4.87	−2.55	28	7.96	21.7	72.3	319.4
Fall	−50.26	−7.80	−4.09	35	12.18	11.9	69.5	189.1
Winter	−71.83	−10.43	−5.48	37	11.59	−1.6	77.1	136.1
Mean	−43.40	−6.61	−3.46	31	9.45	10.8	71.8	920.9

### Isotopic variations of daily and monthly precipitation at different time scales

#### Precipitation isotopic variations (δ^2^H, δ^18^O and δ^17^O) and the influencing factors

**Figure 2:**
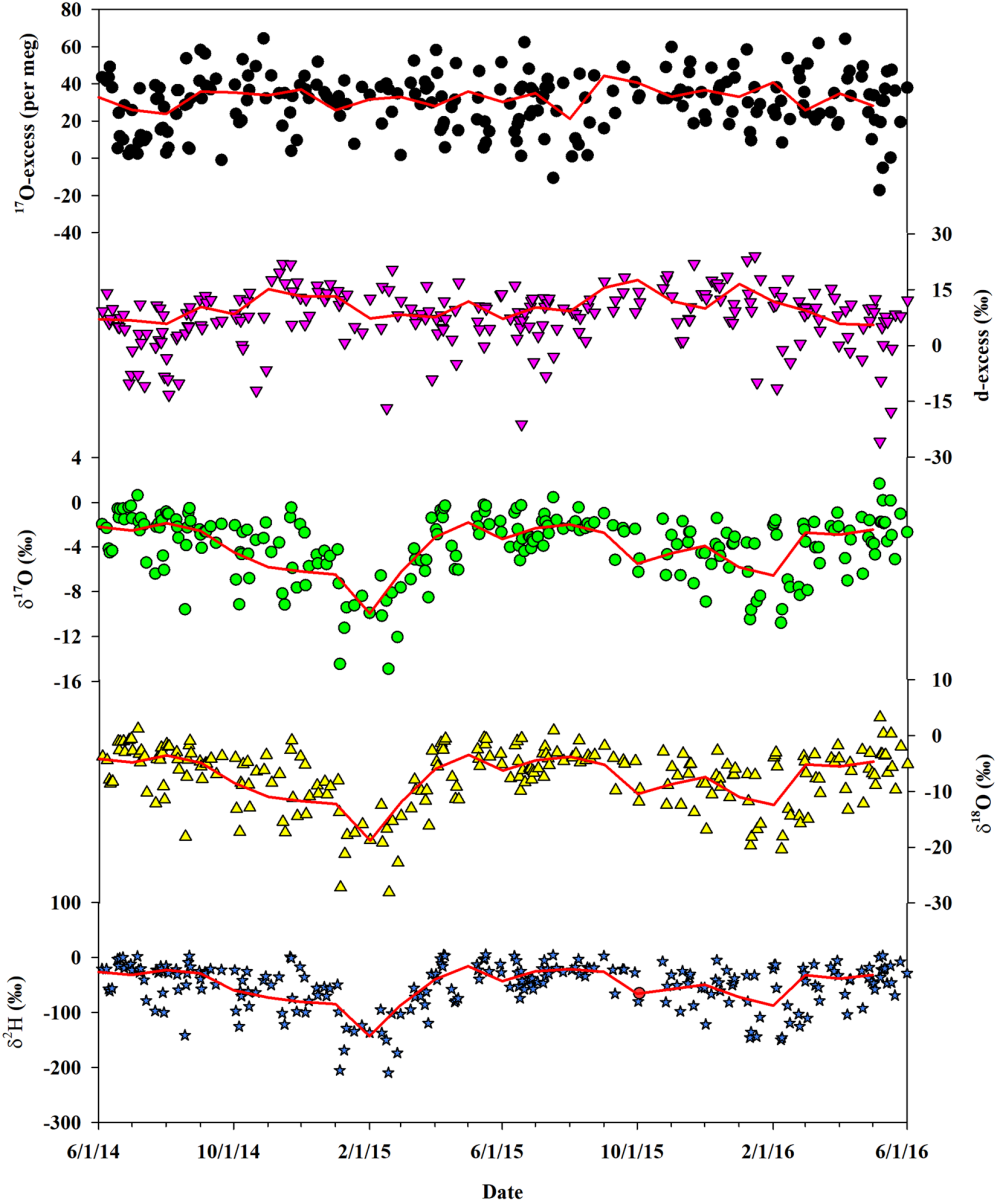
Water stable isotope variations on the event-based sampling (circles, triangles and stars) and corresponding monthly means (red lines) in precipitation between June 2014 and May 2016 in west-central United States. From top to bottom: ^17^O-excess, d-excess, δ^17^O, δ^18^O and δ^2^H during the individual event.

A wide range of the δ^18^O values was observed in the daily precipitation data during the study period (−28.10‰ to 3.23‰) (Fig. 2), which is greater than what has been observed in other Midwest regions (e.g., the Chicago area; −18.37‰ to −3.18‰)^40^. The amount-weighted mean value (−6.54‰) is higher than what observed in Switzerland (−9.05‰) and the continental U.S. (−8.0‰)^9,19^. Large daily precipitation δ^18^O variations were also observed within different seasons, showing the largest amplitude in the winter (−28.10‰ to −2.73‰) likely due to the complicated water vapor source. This resembles the results from Bondville, Illinois, which show that winter precipitation can originate from the continental U.S., Gulf of Mexico, Pacific, and Arctic sources^32^. The smallest amplitude of δ^18^O variations occurred in the fall (−17.35‰ to −0.88‰), which may reflect relatively stable meteorological factors (Fig. 1) and relatively consistent water vapor sources as observed in Bondville (mainly from Pacific and continental sources)^32^. The mean values of the δ^18^O showed a seasonal trend with higher value in the summer (−4.93‰) and lower value in the winter (−10.26‰) (Fig. 2). For the monthly precipitation δ^18^O trends, the mean value over the study period (−6.61‰) as well as the seasonal trend and values were all similar to the daily precipitation values (Table 1). The δ^2^H and δ^17^O variations showed similar trends to δ^18^O for both daily and monthly scales (Fig. 2 and Table 1).

As demonstrated in many previous studies^3,7,9^, local meteorological factors play an important role for precipitation isotopic variations in addition to the moisture source influence. Therefore, to better understand what factors affect precipitation isotopic values over the study period and within different seasons, we investigated their relationships with temperature, RH, and precipitation amount at the site of precipitation. The isotopes (δ^2^H, δ^18^O and δ^17^O) of daily and monthly precipitation over the study period were all affected by temperature (r ≈ 0.71 and 0.88 for daily and monthly precipitation, respectively) (Table 2), which is consistent with the traditional “temperature effect” in subtropical and mid-latitude sites (e.g., in Switzerland (r ≈ 0.56 and 0.85))^9,15^. It is interesting to note that positive correlations between monthly isotopic composition (δ^2^H, δ^18^O and δ^17^O) and precipitation amount (r ≈ 0.53) over the study period were found (Table 2), in contrast to the classic “amount effect” in tropical regions^8,15,43,44^. In fact, greater precipitation often occurred in the summer due to moisture being sourced from the Gulf of Mexico^32,33^, the “anti-amount effect” corresponded to the higher temperature in the summer. We therefore suggest that the observed positive relationship between monthly isotopic composition and precipitation amount is the result of the covariance between isotopes and temperature, and isotope variations are mainly affected by temperature during the study period. Moreover, the spring and winter precipitation isotopic compositions exhibited stronger correlations with daily temperature (r ≈ 0.63 and 0.80) than those observed in the summer and fall (r ≈ 0.35 and 0.44) (Table 3), possibly due to the relatively uniform temperature patterns in the summer and fall (Fig. 1)^45^. The strong correlations and similar sensitivities between monthly isotopic composition and temperature were also observed in the spring, fall and winter (r ≈ 0.85, 0.83 and 0.86) (Table 4). These indicated that aggregation of precipitation isotopic compositions from daily to monthly scale increases the sensitivity to temperature and reduces the difference between seasons. The winter precipitation isotopic compositions were also affected by the daily and monthly RH (r ≈ 0.44 and 0.95) (Tables 3 and 4), which is similar to what reported for a two-year study (monthly scale) in Switzerland (r ≈ 0.40)^9^.

**Table 2:** The relationships between the precipitation isotopes (δ^2^H/δ^18^O/δ^17^O), ^17^O-excess, d-excess and local meteorological parameters (temperature (T), relative humidity (RH) and precipitation amount (P)) at both daily and monthly time scales over the study period.

	δ^2^H		δ^18^O		δ^17^O		^17^O-excess		d-excess	
	r	*p*	r	*p*	r	*p*	r	*p*	r	*p*
T_daily_	0.69	<0.001	0.71	<0.001	0.71	<0.001	−0.14	0.032	−0.20	0.003
RH_daily_	—	—	—	—	—	—	—	—	0.20	0.002
P_daily_	—	—	—	—	—	—	0.17	0.022	0.20	0.005
T_monthly_	0.87	<0.001	0.88	<0.001	0.88	<0.001	—	—	—	—
RH_monthly_	—	—	—	—	—	—	—	—	—	—
P_monthly_	0.51	0.012	0.54	0.007	0.54	0.007	—	—	−0.41	0.046

“−” indicates insignificant correlation.

**Table 3:** The relationships between the daily precipitation isotopes (δ^2^H/δ^18^O/δ^17^O), ^17^O-excess, d-excess and local meteorological parameters (temperature (T), relative humidity (RH) and precipitation amount (P)) within different seasons.

	δ^2^H		δ^18^O		δ^17^O		^17^O-excess		d-excess	
	r	*p*	r	*p*	r	*p*	r	*p*	r	*p*
T_spring_	0.65	<0.001	0.62	<0.001	0.62	<0.001	—	—	—	—
RH_spring_	— — — —									
P_spring_	—	—	—	—	—	—	—	—	0.26	0.041
T_summer_	0.35	0.003	0.35	0.004	0.33	0.004	−0.26	0.023	—	—
RH_summer_	— — — — —									
P_summer_	—	—	—	—	—	—	0.28	0.032	0.37	0.003
T_fall_	0.42	0.003	0.45	0.002	0.45	0.001	0.35	0.015	—	—
RH_fall_	— — — — —									
P_fall_	— — — — —									
T_winter_	0.82	<0.001	0.79	<0.001	0.79	<0.001	—	—	0.36	0.009
RH_winter_	0.47	<0.001	0.42	0.002	0.42	0.002	—	—	0.37	0.006
P_winter_	—	—	—	—	—	—	0.32	0.045	—	—

“−” indicates the insignificant correlation.

**Table 4:** The relationships between the monthly precipitation isotopes (δ^2^H/δ^18^O/δ^17^O), ^17^O-excess, d-excess and local meteorological parameters (temperature (T), relative humidity (RH) and precipitation amount (P)) within different seasons.

	δ^2^H		δ^18^O		δ^17^O		^17^O-excess		d-excess	
	r	*p*	r	*p*	r	*p*	r	*p*	r	*p*
T_spring_	0.85	0.030	0.85	0.031	0.85	0.031	—	—	—	—
RH_spring_	— — — — —									
P_spring_	— — — — —									
T_summer_	— — — — —									
RH_summer_	— — — — —									
P_summer_	—	—	—	—	—	—	0.85	0.032	—	—
T_fall_	0.85	0.030	0.82	0.044	0.82	0.044	—	—	—	—
RH_fall_	— — — — —									
P_fall_	—	—	—	—	—	—	—	—	−0.92	0.008
T_winter_	0.84	0.037	0.87	0.024	0.87	0.024	—	—	—	—
RH_winter_	0.91	0.014	0.97	0.009	0.97	0.009	—	—	—	—
P_winter_	— — — — —									

“−” indicates the insignificant correlation.

#### Variations of ^17^O-excess (d-excess)

**Figure 3:**
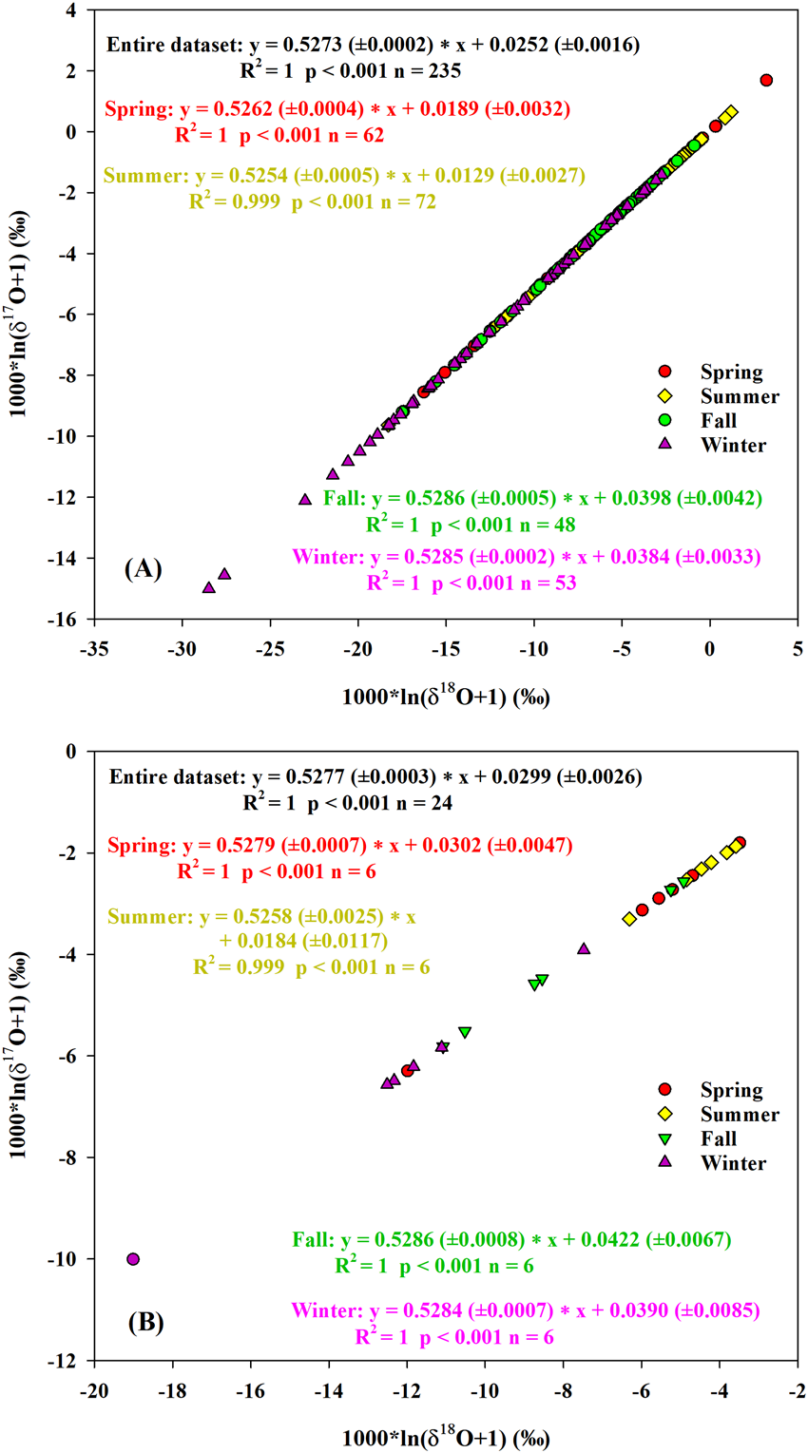
The relationships between δ^17^O and δ^18^O based on daily (**A**) and monthly (**B**) precipitation within different seasons between June 2014 and May 2016.

**Figure 4:**
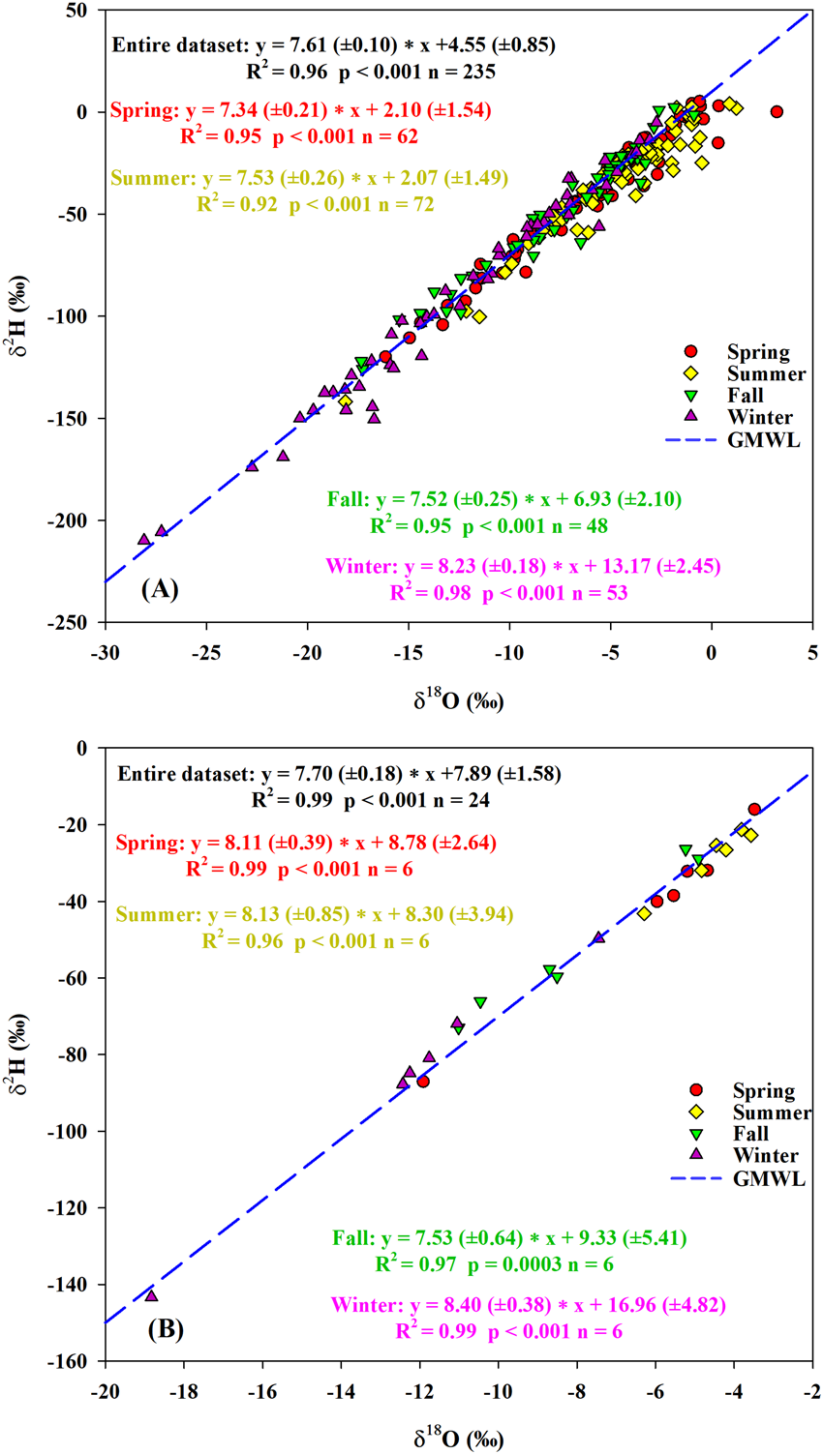
Local meteoric water lines from δ^2^H and δ^18^O in daily (**A**) and monthly (**B**) precipitation within different seasons between June 2014 and May 2016.

^17^O-excess (d-excess) values of daily precipitation during the study period (−17 to 64 per meg (–25.79‰ to 24.02‰)) (Fig. 2) are comparable to what is obtained from Switzerland (−26 to 72 per meg (−27.96‰ to 21.95‰))^9^, while the range is larger than what has been observed across the continental U.S. (tap water, −6 to 43 per meg (−2.5‰ to 17.8‰))^19^. The mean value of ^17^O-excess (d-excess) (31 per meg (9.40‰)) is close to the global meteoric waters (35 ± 16 per meg (10‰))^19,29^, but it is larger than what observed in some mid-latitude regions (e.g., Switzerland (18 per meg)^9^ and the continental U.S. (17 ± 11 per meg)^19^), while lower than that reported from Chicago (59 per meg) where the moisture sources of meteoric waters are the Gulf of Mexico and Lake Michigan leading to higher values^40^. The range of ^17^O-excess values in the spring (−17 to 64 per meg) and summer (−11 to 62 per meg) were larger than during the winter (8 to 58 per meg), which was the opposite trend for the range of seasonal δ^18^O variations (Fig. 2). This indicates that ^17^O-excess brings additional information on precipitation formation, and the moisture source is not the dominant control on winter ^17^O-excess variations. More intra-seasonal variability is observed in our study compared to the African monsoon region where ^17^O-excess remains relatively stable before the monsoon onset and slowly changes during the monsoon season^8^. In addition, mean values of ^17^O-excess (d-excess) at our study site were the lowest in the spring and summer (30 per meg (8.07‰ and 8.06‰)), whereas values in the fall and winter were higher (36 and 34 per meg (12.18‰ and 11.50‰)). The seasonal pattern of ^17^O-excess (d-excess) is similar to the trend reported in Switzerland having the lowest value in summer (13 per meg (~7.1‰)) and the highest in winter (25 per meg (~8.1‰)). This demonstrates that ^17^O-excess variations have obvious seasonal pattern under the relative influence of kinetic and equilibrium fractionations^9^, corresponding to different slopes of δ′^18^O-δ′^17^O (as a proxy of fractionation factor) within different seasons (Fig. 3). d-excess variations indicate less re-evaporation at the precipitation site in the fall and winter, which is also verified by the higher slope and intercept of local meteoric water line (LMWL) between δ^2^H and δ^18^O in the fall (7.52/6.93‰) and winter (8.23/13.17‰) (Fig. 4A). For monthly precipitation, ^17^O-excess values (d-excess) ranged from 21 to 44 per meg (5.55‰ to 17.61‰) during the study period with an average of 31 per meg (9.45‰) (Table 1), similar to the mean value of daily precipitation. The mean value of ^17^O-excess values in the summer was the lowest (28 per meg), and the value in the winter was the highest (37 per meg), showing the similar seasonal variation trend with daily precipitation, and d-excess as well.

#### The relationships between ^17^O-excess, δ^18^O, and d-excess

Owing to the complexity of the moisture source over the two years, we, for the first time, quantitatively analyzed the relationships between ^17^O-excess and both δ^18^O and d-excess of precipitation at different time scales in the mid-latitude region to probe the evaporative conditions at the moisture source. According to the conceptual evaporation model under both steady and non-steady state conditions, if kinetic fractionation associated with evaporation is the sole influencing factor, ^17^O-excess should be anti-correlated with δ^18^O, positively correlated with d-excess, and the slope of the latter should be 0.7–2.0 per meg/‰ (or the slopes of δ′^18^O-δ′^17^O between 0.5183 and 0.5265), which could also reflect RH at the site of evaporation (i.e., at the moisture source)^19^.

**Figure 5:**
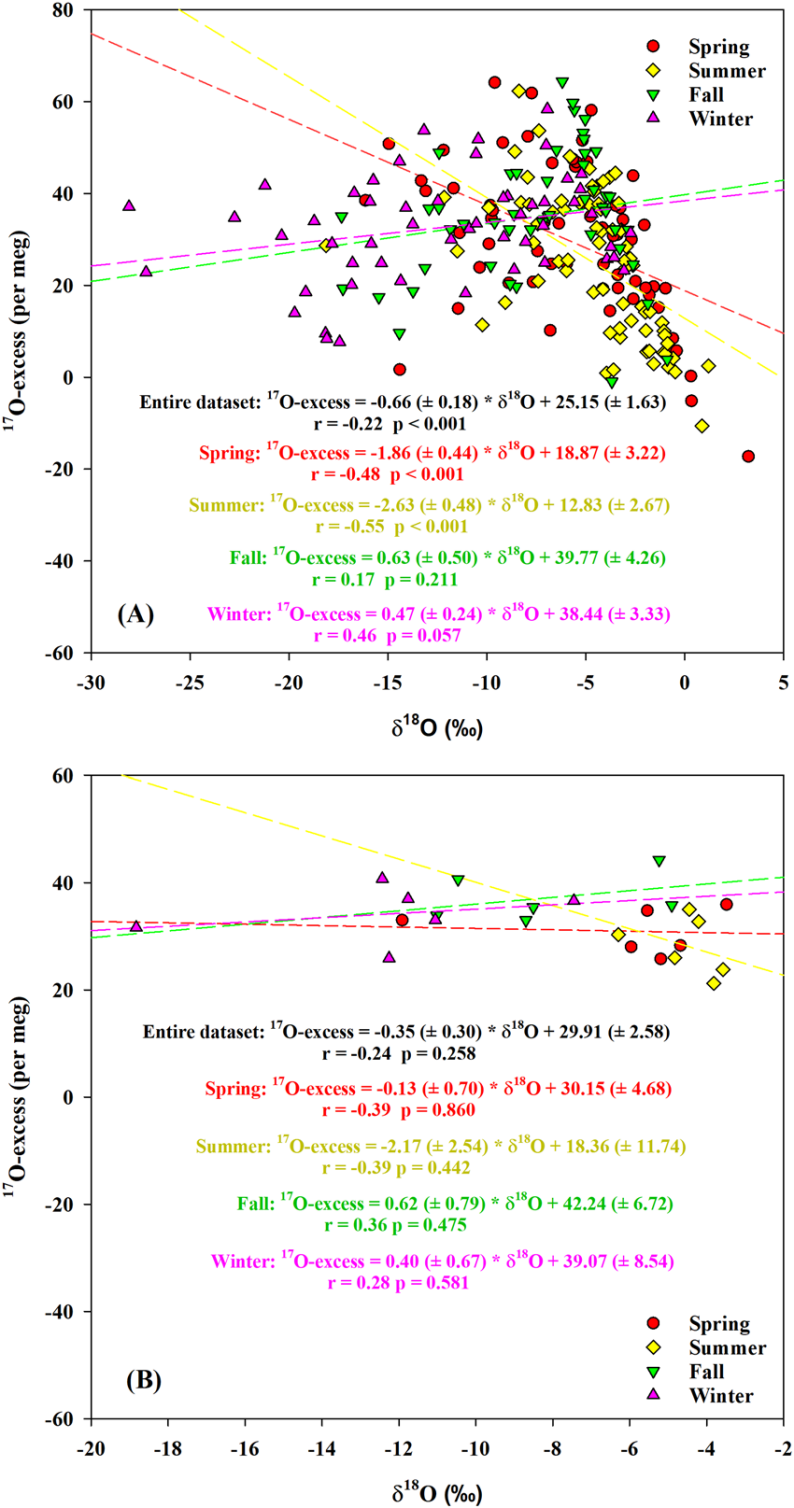
The relationships between ^17^O-excess and δ^18^O based on daily (**A**) and monthly (**B**) precipitation within different seasons between June 2014 and May 2016.

**Figure 6:**
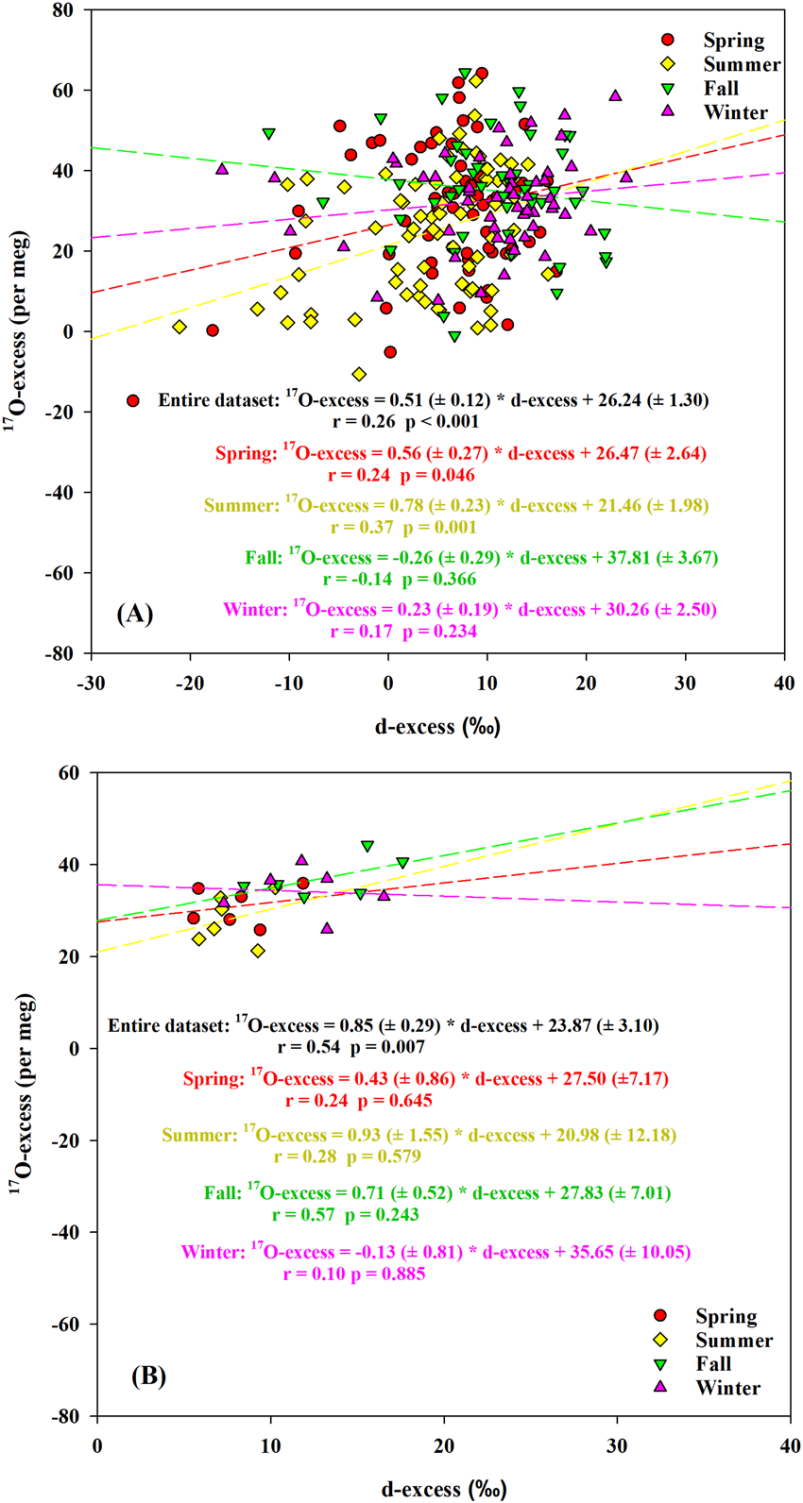
The relationships between ^17^O-excess and d-excess based on daily (**A**) and monthly (**B**) precipitation within different seasons between June 2014 and May 2016.

In our study, the anti-correlation between daily precipitation ^17^O-excess and δ^18^O was weak (r = −0.22, *p* < 0.001) (Fig. 5A) and a weak positive correlation with d-excess was also observed (r = 0.26, *p* < 0.001, slope = 0.51 ± 0.12 per meg/‰) (Fig. 6A) over the study period. This suggests that multiple factors, in addition to the kinetic fractionation effect associated with evaporation at the oceanic source regions, influence precipitation isotopic compositions^1,14,19,29^. In addition, daily precipitation ^17^O-excess was anti-correlated to δ^18^O in the spring (r = −0.48, *p* < 0.001) and summer (r = −0.55, *p* < 0.001) (Fig. 5A). The δ′^18^O-δ′^17^O slopes of daily precipitation were 0.5262 (±0.0004) and 0.5254 (±0.0005) for the spring and summer, respectively (Fig. 3A), which are close to the slopes of tap waters (as a proxy of precipitation) from the eastern and western U.S. (0.526–0.527)^19^. Based on theoretical predictions^19^, these results suggest that precipitation experienced steady-state evaporation processes with RH between 50% and 85% in the spring and summer leading to the lower ^17^O-excess. The positive correlation between ^17^O-excess and d-excess for summer daily precipitation (r = 0.37, *p* = 0.001, slope = 0.78 ( ± 0.23) per meg/‰) (Fig. 6A) is within the range of the slopes for the theoretical relationships (0.7–2.0 per meg/‰) as mentioned above, further supporting the steady-state kinetic fractionation effect. The slope is similar to what is observed in Africa (0.94–1.04 per meg/‰), where the precipitation experiences steady-state re-evaporation and convective processes^8^. However, the slope is different from the Gulf states (the most southerly U.S.; 2.5 ± 1.2 per meg/‰), where the precipitation experiences non steady-state re-evaporation processes^19^. It is interesting that the slope of δ′^18^O-δ′^17^O for daily precipitation in the fall (0.5286 ± 0.0005) was close to that in the winter (0.5285 ± 0.0002) (Fig. 3A). Both were similar to the equilibrium fractionation coefficient of the vapor-liquid equilibrium during 10–40 °C (0.529 ± 0.001)^39^ and the vapor-solid equilibrium between 0 °C and −40 °C (0.5285–0.5290)^46^. Similar slopes are observed in Chicago precipitation (0.529 ± 0.003) and NEEM (Greenland) snow (0.528 ± 0.001)^27,40^. In addition, the lower slope in the summer than winter is similar to the result from Switzerland (0.5255 ± 0.0009 and 0.5271 ± 0.0004 for summer and winter, respectively)^9^, which is due to higher kinetic fractionation processes in the summer. There were no relationships of monthly precipitation between ^17^O-excess and both δ^18^O and d-excess within different seasons (*p* > 0.05) (Figs. 5B and 6B). Moreover, the δ′^18^O-δ′^17^O slope of monthly precipitation in the spring (0.5279 (±0.0007) was obviously higher than that of the daily one (Fig. 3B). These demonstrate that only daily precipitation isotopic variations could better reflect evaporation information at the moisture source, and monthly aggregation loses some evaporation information.

#### Local factors influencing d-excess and ^17^O-excess

^17^O-excess variations reflect different precipitation formation mechanisms at the precipitation site for different latitudes, such as raindrop re-evaporation effect (positive correlation with RH) in Africa^8^, solid condensation under supersaturation (positive correlation with temperature) in the polar regions^10,25^, and negative correlation with temperature in the mid-latitude^9^. Therefore, to better identify the influencing factors of d-excess and ^17^O-excess at the site of precipitation, it is necessary to disentangle their sensitivity to temperature, RH, and precipitation amount over the study period and within different seasons. We found that the d-excess and ^17^O-excess of daily precipitation over the study period were almost not affected by the local meteorological factors due to the weak correlations (r < = 0.20) (Table 2). However, 41% of variance in d-excess was explained by the monthly precipitation amount (Table 2), similar to what obtained from Switzerland (39%)^9^. In addition, for d-excess and ^17^O-excess in precipitation (daily or monthly) over the study period, d-excess has relatively stronger correlations with temperature, RH, and precipitation amount than does ^17^O-excess (Table 2). Therefore, relative to the d-excess, precipitation ^17^O-excess over the study period were mainly affected by the atmospheric conditions at the moisture source and along moisture transport trajectories, while almost not affected by the local meteorological factors. This is different from what is observed in Switzerland and it is found ^17^O-excess contains the information about local monthly temperature^9^.

Sensitivities of d-excess and ^17^O-excess to the local meteorological factors within seasons seem to be more complex than over the whole study period. In the summer, d-excess correlated positively with the daily precipitation amount (r = 0.37) (Table 3), similar to what observed in Africa reflecting the “amount effect”, caused by re-evaporation of raindrops at the precipitation site^8^. In the winter, d-excess was slightly affected by both the daily temperature and RH (r = 0.36/0.37) (Table 3). Only the d-excess in Africa is significantly correlated with RH both at the seasonal scale and during convective processes (r = 0.82/0.90), which indicates re-evaporation is a key controlling process due to the importance of RH in evaporation models^8^. This means that d-excess in our winter precipitation might be affected by thunderstorms during warm winters (mean temperature was 0.3 °C and the range was −13.3 to 15.6 °C over the two winters) and the associated high RH (83%; 62–96%) (e.g., the thunderstorm occurred on Dec 23^th^ 2015 and the temperature and RH were 11.1 °C and 93%, respectively). In addition, d-excess values in the fall exhibited a strong negative correlation with the monthly precipitation amount (r = −0.92) (Table 4), showing the opposite trend of summer daily precipitation.

It is worth noting that precipitation ^17^O-excess (daily or monthly) was not affected by local meteorological factors in the spring (*p* > 0.05) (Tables 3 and 4), therefore, ^17^O-excess in the spring could be used as a tracer of the evaporative conditions at the moisture source. However, ^17^O-excess in other seasons was affected by different meteorological factors. For example, ^17^O-excess in the fall was positively correlated with the local daily temperature (r = 0.35) (Table 3), while a negative correlation was observed in Switzerland based on monthly temperature from 2012–2014 (r = −0.45)^9^. Only Antarctica shows positive correlations between snow ^17^O-excess and temperature due to the kinetic fractionation under supersaturation^10,25,26^. In fact, the fall precipitation is unlikely to be caused by supersaturation due to the higher temperature range (−7 to 27 °C) compared to polar region and abundant condensation nuclei in the mid-latitudes, while higher ^17^O-excess with high temperature might be due to the continental recycling of the moisture in the fall^19^. Moreover, a positive relationship was observed between ^17^O-excess and the daily precipitation amount in the winter (r = 0.32) (Table 3), and the sensitivity was far less than that for monthly precipitation amount in the summer (r = 0.85) (Table 4), demonstrating that the ^17^O-excess in the winter is less affected by kinetic fractionation than in the summer. In addition, the relationship between ^17^O-excess in the summer and the monthly precipitation amount is consistent with the trend observed in the Gulf region (r = 0.59, *p* = 0.05) and in the tropics^14,19^. The amount effect suggests that the precipitation ^17^O-excess in the summer may have been affected by stronger kinetic fractionation associated with re-evaporation of raindrops at the precipitation site^47^. Convective processes should also be considered especially for gentle thunderstorm events, giving rise to lower ^17^O-excess in the summer (e.g., on July 17, 2015 (−11 per meg) and May 7, 2016 (−17 per meg)), which is similar to what observed in the central U.S.^19^.

### The isotopic characteristics in rainfall and snowfall

As far as we know, there is no previous research on the difference between rainfall and snowfall isotopes in the mid-latitudes. Additionally, previous studies of snow isotopes (e.g., snowfall, snow pits and ice cores) have mainly focused on the polar regions. However, even under similar lower temperature conditions the isotope variations are sensitive to different mechanisms^10,25,27^. Therefore, to better understand the precipitation mechanisms, it is necessary to study the different forms of precipitation isotopes in the east-central U.S. and compare the snowfall isotopes variations between mid-latitudes and high-latitudes.

**Figure 7:**
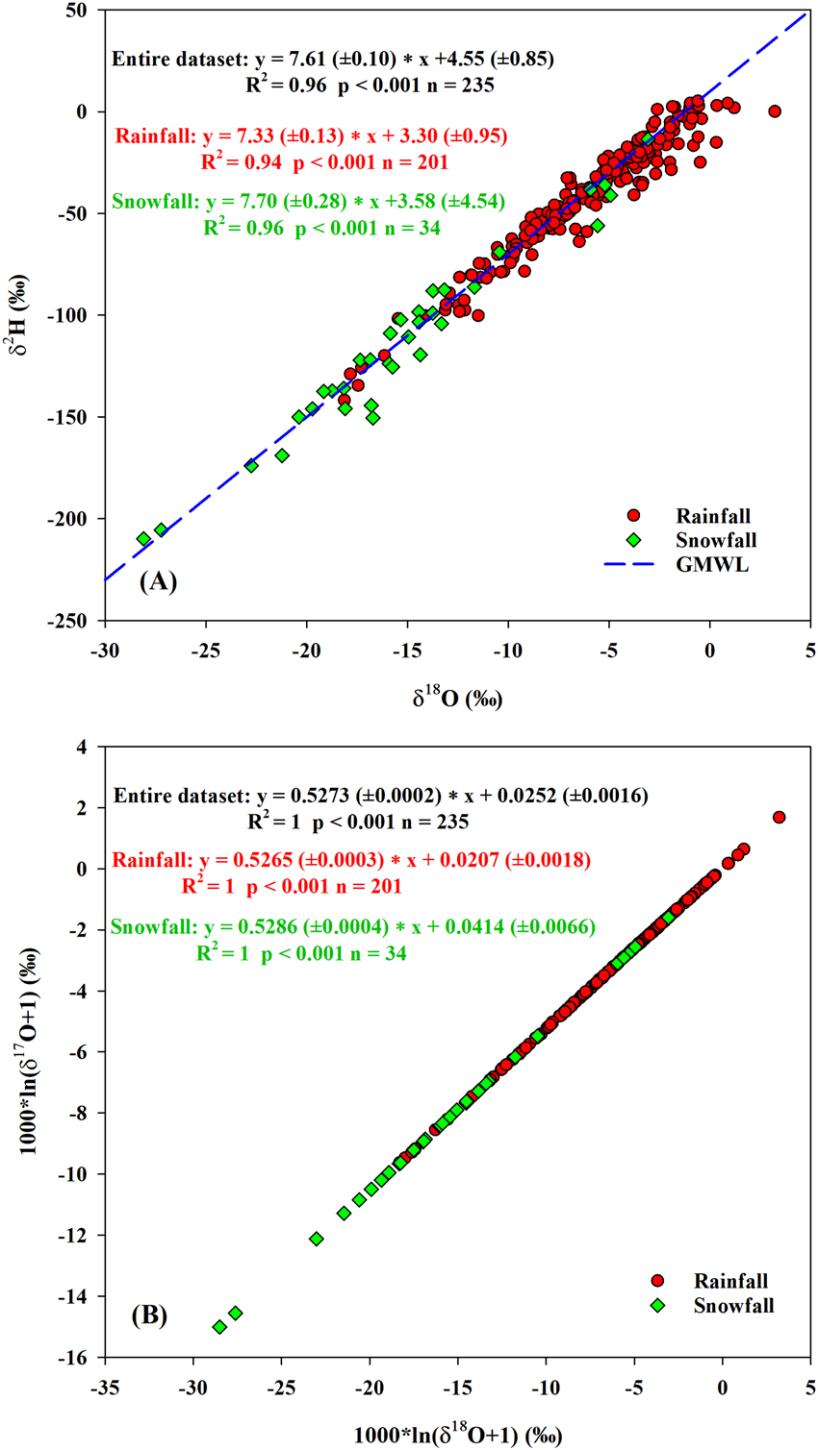
The relationships between δ^18^O and δ^2^H (**A**), δ^18^O and δ^17^O (**B**) in rainfall and snowfall between June 2014 and May 2016.

The large range (−28.10‰ to −3.07‰) and depleted average value (−13.05‰) of snowfall δ^18^O, compared with those of rainfall in the site (−18.13‰ to 3.23‰; −6.10‰), may be due to lower temperatures, different air mass trajectories and different moisture source regions^28^. The slope and intercept of local meteoric water line (LMWL) between δ^2^H and δ^18^O for the rainfall (7.33/3.30‰) were both lower than those of the snowfall (7.70/3.58‰) (Fig. 7A), showing stronger re-evaporation effect for rainfall. Most of snow δ^18^O values in the high-latitudes (e.g., Alert Canada (−39.4‰ to −34.5‰) and Vostok (−60‰ to −50‰)) are lower than ours, possibly due to the polar climate^12,40^. In addition, δ^2^H, δ^18^O and δ^17^O in our study were sensitive to the daily temperature regardless of rainfall and snowfall (Table 5), and snowfall variations were more sensitive to the temperature (r ≈ 0.73) than the rainfall (r ≈ 0.48), which is also observed between ice cores δ^18^O and local temperature in Dome A (−60 to −15 °C) and Vostok (−32 to −38 °C), Antarctic^13,25^.

**Table 5:** The relationships between the precipitation isotopes (δ^2^H/δ^18^O/δ^17^O), ^17^O-excess, d-excess and local meteorological parameters (temperature (T), relative humidity (RH) and precipitation amount (P)) for rainfall and snowfall over the study period.

	δ^2^H		δ^18^O		δ^17^O		^17^O-excess		d-excess	
	r	*p*	r	*p*	r	*p*	r	*p*	r	*p*
T_rainfall_	0.45	<0.001	0.49	<0.001	0.49	<0.001	−0.20	0.007	−0.28	<0.001
RH_rainfall_	—	—	—	—	—	—	—	—	0.20	0.006
P_rainfall_	—	—	—	—	—	—	0.17	0.034	0.22	0.004
T_snowfall_	0.75	<0.001	0.71	<0.001	0.71	<0.001	—	—	—	—
RH_snowfall_	— — — — —									
P_snowfall_	0.44	0.033	—	—	—	—	—	—	—	—

“−” indicates the insignificant correlation.

For ^17^O-excess, the range of values in rainfall (−17 to 64 per meg) was greater than those for snowfall (2 to 54 per meg). The average of snowfall (34 per meg) was slightly higher than that of rainfall (32 per meg), and both are close to the global meteoric waters (35 ± 16 per meg)^19,29^. The ^17^O-excess range of rainfall in our study is much larger than what is obtained from African monsoon precipitation (−10 to 20 per meg) experiencing much stronger raindrop re-evaporation^8^. In addition, the ^17^O-excess variations of snowfall in this Midwest region seem to be less complex compared with previous studies in polar regions. For example, our ^17^O-excess average of snowfall is close to what observed in the Greenland snow (35 ± 13 per meg)^27^. However, the snowfall ^17^O-excess range varied considerably from 9 to 51 per meg along the East Antarctica traverse, showing a significant decreasing trend along the traverse due to the difference in supersaturation along the air mass trajectories at low temperature^25^. Although our snowfall ^17^O-excess range is comparable to them, the snowfall in our site were actually not affected by the supersaturation which would be explained further below.

**Figure 8:**
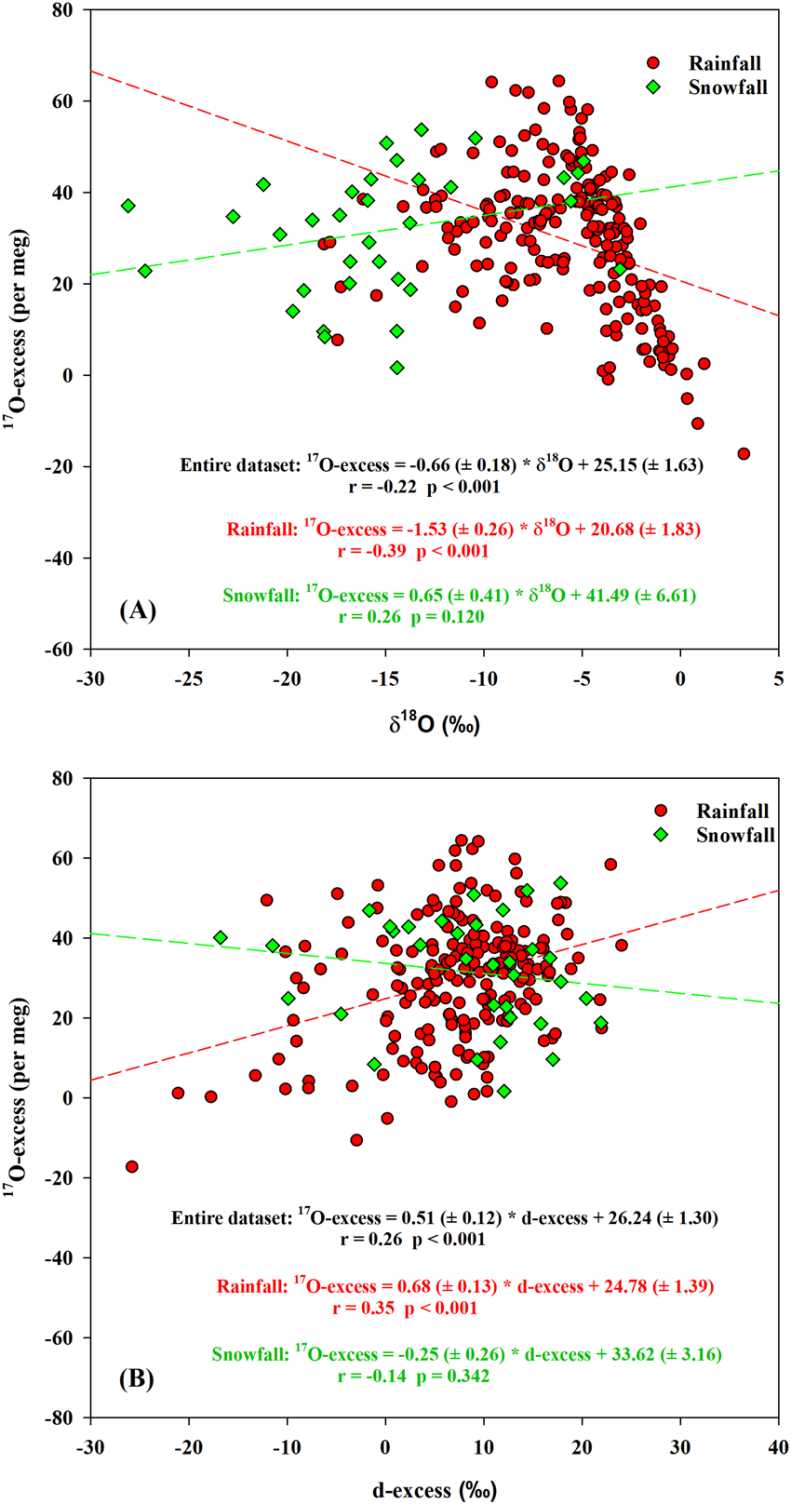
The relationships between ^17^O-excess and both δ^18^O (**A**) and d-excess (**B**) for rainfall and snowfall between June 2014 and May 2016.

Similar to the analyses of precipitation at different time scales, we also inferred the fractionation differences at the moisture source between rainfall and snowfall through the conceptual evaporation model. The rainfall ^17^O-excess exhibited a negative correlation with δ^18^O (r = −0.39, *p* < 0.001) and a positive correlation with d-excess (r = 0.35, *p* < 0.001, slope = 0.68 ( ± 0.13) per meg/‰), while no correlations were observed in snowfall (Fig. 8). This indicates that snowfall is not affected by supersaturation since a positive correlation between ^17^O-excess and δ^18^O should appear under supersaturation such as those in Vostok snowfall^10^. In addition, the rainfall δ′^18^O-δ′^17^O slope (0.5265 (±0.0003)) was lower than in snowfall (0.5286 (±0.0004)) (Fig. 7B). The slope of snowfall is close to the equilibrium fractionation coefficient for vapor-solid equilibrium during 0 °C and −40 °C (0.5285–0.5290)^46^. Our results indicate that the rainfall is affected by the kinetic fractionation during steady-state evaporation processes at the moisture source, while the snowfall seems to be more affected by equilibrium fractionation.

The correlation analyses showed that the rainfall ^17^O-excess and d-excess were not influenced by the local meteorological factors indicated by the weak correlations (|r|< = 0.28) (Table 5), which were slightly different from the precipitation seasonal sensitivities. Compared with rainfall d-excess, ^17^O-excess were less affected by the local meteorological factors. In addition, there were no correlations between the snowfall ^17^O-excess and the local meteorological factors (*p* > 0.05). Therefore, ^17^O-excess in rainfall and snowfall could be considered as tracers of evaporative conditions at the moisture source in present study.

## Conclusions

Ground-based precipitation isotope records in the mid-latitudes, including the U.S. Midwest, are rare and detailed ^17^O-excess data from the mid-latitude regions is not seen in literature. To fill these knowledge gaps, the isotopic compositions of event-based precipitation including both rainfall and snowfall were monitored at a site in the west-central U.S. The precipitation δ^2^H, δ^18^O and δ^17^O variations were mainly influenced by temperature over the study period. Based on the conceptual evaporation model, the relationships between ^17^O-excess and both δ^18^O and d-excess (or δ′^18^O-δ′^17^O) indicated that the precipitation in the spring and summer experienced steady-state kinetic fractionation during evaporation at the moisture source, as well as for the rainfall (vs snowfall). The precipitation in the fall and winter, as well as for the snowfall, were mainly affected by the equilibrium fractionation. The precipitation ^17^O-excess was affected by some local meteorological factors at the seasonal scale (e.g., monthly precipitation amount in the summer) except in the spring. However, ^17^O-excess of the rainfall and snowfall were not affected by the meteorological factors over the whole study period. Consequently, ^17^O-excess of rainfall, snowfall and the spring precipitation could be considered as tracers of evaporative conditions at the moisture source in this Midwestern site. The precipitation ^17^O-excess at different temporal scales provides additional information to better understand the precipitation formation processes in the mid-latitude regions.

## Electronic supplementary material

Supplementary Information
