# Supplementary material for: Stable isotope compositions (δ2H, δ18O and δ17O) of rainfall and snowfall in the central United States
Source: Sci Rep. 2018 Apr 30;8:6712. doi: 10.1038/s41598-018-25102-7 (PMC5928101; doi:10.1038/s41598-018-25102-7)
Supplement: Supplementary file 1 — Supplementary Information [file 41598_2018_25102_MOESM1_ESM.pdf]

Stable isotope compositions (δ^2H, $\delta^{18}O$ and $\delta^{17}O$) of rainfall and snowfall in the central United States

Chao Tian, Lixin Wang*, Kudzai Farai Kaseke and Broxton W. Bird

Department of Earth Sciences, Indiana University-Purdue University Indianapolis (IUPUI), IN 46202, USA

***Corresponding author**
Lixin Wang
Department of Earth Sciences
Indiana University-Purdue University Indianapolis
Indianapolis, IN, 46202, USA
Office phone number: 317-274-7764
Email: lxwang@iupui.edu

Supplementary data

Table S1. Summary of the precisions of δ^2H, $\delta^{18}O$, $\delta^{17}O$ and ^{17}O-excess for two international standards (SLAP and GISP) and five commercially available working standards from Los Gatos Research Inc. (LGR; Mountain View, CA, USA).

	Precision			
**Samples**	**δ^2H (‰)**	**$\delta^{18}O$ (‰)**	**$\delta^{17}O$ (‰)**	**^{17}O-excess (per meg)**
**$SLAP_{VSMOW}$**	**0.79**	**0.04**	**0.02**	**3**
**$GISP_{VSMOW-SLAP}$**	**0.12**	**0.02**	**0.02**	**7**
**$1A_{VSMOW-SLAP}$**	**0.80**	**0.06**	**0.03**	**8**
**$2A_{VSMOW-SLAP}$**	**0.73**	**0.06**	**0.03**	**2**
**$3A_{VSMOW-SLAP}$**	**0.33**	**0.05**	**0.03**	**12**
**$4A_{VSMOW-SLAP}$**	**0.07**	**0.06**	**0.02**	**8**
**$5A_{VSMOW-SLAP}$**	**0.64**	**0.06**	**0.03**	**5**
